# “It Changed Everything”: Challenges to Indigenous Recovery Practices Amid the COVID-19 Pandemic

**DOI:** 10.3390/genealogy9040105

**Published:** 2025-10-01

**Authors:** Melinda S. Smith, Andria B. Begay, Chesleigh Keene, Alisse Ali-Joseph, Carol Goldtooth-Begay, Manley A. Begay, Juliette Roddy

**Affiliations:** 1 Center for Community Health and Engaged Research, Northern Arizona University, Flagstaff, AZ 86011, USA; 2 Department of Applied Indigenous Studies, Northern Arizona University, Flagstaff, AZ 86011, USA; 3 The Partnership for Native American Cancer Prevention, Northern Arizona University, Flagstaff, AZ 86011, USA; 4 Department of Criminology and Criminal Justice, Northern Arizona University, Flagstaff, AZ 86011, USA

**Keywords:** Indigenous, American Indians, Native Americans, COVID-19, substance use recovery, addiction treatment, qualitative

## Abstract

**Background:**

The COVID-19 pandemic exacerbated existing health inequities for Native American communities, intensifying the challenges faced in accessing addiction and recovery services. As part of a tribal-university collaborative effort in Arizona, our team explored the impacts of the COVID-19 pandemic on mental well-being and resilience among the Indigenous substance use recovery community.

**Methods:**

We conducted qualitative analysis of transcribed individual interviews (*n* = 19) to understand the factors of resilience and mental well-being for providers of Western addiction treatment services and Indigenous community members who were in addiction recovery or engaged in addiction treatment during the pandemic.

**Results:**

Four major themes that impacted mental well-being among the Indigenous recovery group during the pandemic were identified: (1) healthcare barriers; (2) culture in recovery; (3) the impact of colonization/historical trauma; and (4) the importance of relationships.

**Conclusions:**

This work provides insight into the disproportionate impact of the COVID-19 pandemic on Indigenous communities and vulnerable populations such as the recovery community. Findings from this study highlight the need for Indigenous-grounded and culturally informed recovery interventions.

## 1. Introduction

The COVID-19 pandemic exacerbated challenges in substance use treatment and addiction recovery service among American Indians (AI)/Indigenous Peoples, underscoring the deep-rooted health disparities that have persisted. Nationwide, drug overdose deaths surged by nearly 30% from 2019 to 2020; the largest increases occurred among Black (44%) and American Indian and Alaska Native (AI/AN) (39%) populations (Kariisa 2022). The pandemic disrupted public health efforts and societal norms on a global scale, and its impact on mental health and well-being remains incompletely understood.

For Indigenous communities across the United States (US), the COVID-19 virus compounded existing vulnerabilities, further marginalizing an already disproportionately affected population and widening the health inequity gap (Lopez 2020; Wong et al. 2022). Research shows that AI populations exhibit high rates of abstinence from alcohol use compared to other racial groups in the country, but the enduring consequences of colonization have led to disproportionately high rates of substance use disorders (SUD), affecting generations of Indigenous individuals, families, and communities (Skewes et al. 2024). With the onset of COVID-19, individuals in the recovery community experienced a disruption in Western SUD treatment and services (Mellis et al. 2021), increased stress due to the uncertainty that the virus caused (Hansel et al. 2022), increased death rates from substance use (Roberts et al. 2021), and increased financial uncertainty coupled with decreased social services, in general (Conway et al. 2022).

In addition to the COVID-19 pandemic impacting SUD services and treatment, it further exposed and intensified the health inequities that are deeply rooted in the historical systemic oppressions perpetuated by the US against AI communities (Wong et al. 2022). Indigenous Peoples have resiliently endured the political ailments, criminalization, discrimination, and stigmatization that came with the pandemic. Such actions, stemming from the historical and ongoing colonization, have understandably influenced AI communities’ general health and well-being (Roach and McMillan 2022). However, Indigenous Peoples have demonstrated cultural strengths in their pandemic response (Foxworth et al. 2021) through strategic implementations of vaccination strategies to community-wide lockdowns and community curfews to combat the pandemic and protect the health and well-being of community members (Burki 2021).

This qualitative exploration is part of a larger study that started in 2020 (Baldwin et al. 2023), conducted with four community groups (first responders, educators, traditional knowledge holders and practitioners, and members of the substance use recovery community) working in or near three Indigenous Nations in Arizona. This study was initiated in response to concerns raised by community partners about the pandemic’s growing impact on mental health, wellness, and recovery efforts. The purpose of the study was to investigate the impacts of the COVID-19 pandemic on mental health, well-being, and resilience among the four community groups. The overarching research questions that guided this study included: (1) What role does Indigenous Determinants of Health (IDOH) play in tribal government policies and actions that support Indigenous mental health, well-being, and resilience during the COVID-19 crisis, and (2) How has IDOH impacted Indigenous mental health, well-being, and resilience among first responders, educators, traditional knowledge holders and practitioners, and the substance use recovery community living or working (or both) in or near three Indigenous Nations in Arizona (Baldwin et al. 2023)? This article focuses specifically on the substance use recovery community, including of Indigenous individuals working in or engaged with recovery. Our aim was to center Indigenous voices and provide community-grounded insight into the pandemic’s disruption of recovery services and support systems. It presents the qualitative results that aid in understanding the lived experiences of the Indigenous substance use recovery community and the impact of the COVID-19 pandemic on their mental health, well-being, and resilience. This work provides Indigenous-focused content to amplify Indigenous voices and combat the historically deficit-based narrative that has created a negative image of AI Peoples in the substance use recovery community (Cunningham et al. 2016). Indigenous frameworks of recovery emphasize resilience, cultural empowerment, and community-based strengths, which offer critical perspectives on healing that extend beyond Western models (Jardine and Bourassa 2021). Indigenous resilience encompasses not only individual coping strategies, but also emphasizes cultural continuity, relational accountability, and strength-based approaches rooted in land, ceremony, and language (Jardine and Bourassa 2021). This concept framed our analysis and interpretation of participants’ narratives. Specifically, we present findings from interviews with members of the substance use recovery community about the impact of COVID-19 on mental well-being and substance use, as well as the impact of social restrictions on recovery.

## 2. Results

A total of 19 individuals representing the recovery community participated in semi-structured interviews and focus groups from the three participating Indigenous Nations. Table 1 provides additional demographic information on the participants. Four themes emerged from the qualitative data analysis, including (1) healthcare barriers, (2) culture in recovery, (3) the impact of colonization/historical trauma, and (4) the importance of relationships.

### 2.1. Theme 1: Healthcare Barriers

Access to healthcare and mental health resources significantly supports recovery (Kadir and Fenton 2021). However, healthcare barriers was the first major theme that emerged from the interviews. In response to the COVID-19 pandemic, SUD treatment/recovery providers altered their treatment protocols and how they worked with clients to protect themselves and the community. Consequently, recovery treatment services were significantly disrupted. Some recovery providers articulated that their services were greatly modified and required to move to online formats and reported they faced many challenges.

We’ve had people who had to do phone counseling. So, you missed 80 percent of the communication. Right, because you can’t see their face.

Another provider expressed frustration that the transition to online sessions felt cold and distant, especially when SUD treatment and recovery rely heavily on in-person services and counseling groups. Due to social isolation mandates, providers struggled to provide comfort and support, and the aloofness that came with online meetings strained relationships with clients.

This is a very cold environment being on Zoom. This is not warm. If you go into a room with people who are feeling or emoting and crying or expressing how they feel, you can feel that vicariously when you’re in the room with somebody. When you’re on Zoom, it’s a very…cold kind of interaction. So, I think we’ve lost a lot of people through that.

The recovery community deeply felt the disruption to healthcare services during the pandemic. Many participants shared how the limited access to mental healthcare resources negatively impacted their mental well-being and elevated risks to maintain recovery. For instance, a participant stated,

I can see why a lot of people went through what they went through. I can see exactly why a lot of people lost services at that time because we had to shut down a lot of our services. And I can see why people out there, resorted back to substance abuse, ended up back into domestic violence, cooped up at home with their loved ones and they don’t have the skills and tools to work through the things that they were going through.

### 2.2. Culture in Recovery

The second theme from the interviews was culture in recovery. Through an Indigenous lens, culture is innate to one’s being and works cohesively with the recovery and healing process. During the pandemic, culturally responsive treatment in recovery became more elusive. Due to the intricate and inherent relationship between Indigenous culture and everyday life, culture was not explicitly discussed as a modality for recovery. Instead, culture was viewed as a parallel healing method happening outside of Western concepts of recovery. Participants described culture as foundational to their ways of being and identity.

I saw this book in our book case one day and it was called ‘The Red Road of Wellbriety.’… from Don Cornelius. And so, he has this movement of wellbriety, and a lot of people were like…what does that mean?… So I was reading the books off the shelf [and] this particular book cover caught my eye… and I identified with it and I opened it and then I read [the] preface and I was like… this is you, this is what your parents shared and taught you, this…has been part of your life this whole time.

While culturally responsive treatment was seemingly lacking in recovery services during the pandemic, providers acknowledged the power of community and cultural identification for clients. For example, an Indigenous recovery treatment provider stated,

Just remembering our teachings and doing our own ceremonies for our own smokes, our own prayers and knowing that others were doing this as well. I think we all together [are] reaching the same goal, which was to all stick together and come out of this much better than we might have hoped for.

However, in some instances, culture in recovery was put on the back burner. Recovery practitioners described how the chaos of the pandemic brought additional stressors, and they felt overwhelmed, especially with the transition to tele-behavioral health. Note that cultural practice and ceremony are often performed when one is in a good place, and Indigenous SUD treatment/recovery practitioners may have yet to feel they hold the right state of mind and mental well-being to offer cultural teachings or practices within their recovery services. Providers were not applying cultural practices and traditional knowledge for their clients as they typically would. The following quote reveals a practitioner struggling with their own mental well-being.

It definitely affected my mental health, because it’s frustrating. You know, why do things have to be like this? How come it has to be us, that’s going through this in this way.

While specific aspects of culture were not experienced in recovery treatment, participants shared how their ingrained connection to culture made them resilient.

I don’t want to say that COVID affected us… I feel like it [made] us stronger. Our resilience became much more stronger because now it was something like we’re not only fighting for our lives, we are fighting for so much… we don’t want to lose this culture and this language because those were the elders that we lost who were the teachers of the language, the teachers of the culture, the teachers that held those medicines for us… we were supposed to listen.

### 2.3. Theme 3: The Impact of Colonization and Historical Trauma

Historical and ongoing attempts at Indigenous erasure have lasting consequences that continue to harm and challenge Indigenous communities today. The third identified theme acknowledges the impact of colonization and historical trauma as contributing factors to both substance abuse and the lack of culturally responsive recovery resources. Indigenous Peoples have long endured the maltreatment of colonial efforts to eradicate Indigenous ways of living and being, creating apprehensions and distrust of government and Western-oriented practices. A recovery provider explained,

I think that would be one of the biggest ones, is just that we were doing pretty… we’re getting better with participating in counseling and recovery… for our recovery. But, because of the pandemic and everything kind of being pretty much being on hold for, especially while, you know, we’re getting back up now, but of course, for at least half a year, you know, there was not much… not much services. So, I think that set everybody back. And just kind of maybe promoted that distrust again… that we always have as Native people because of our history with the historical trauma and so forth… that it’s hard for us to form those relationships and get them going.

Some participants shared how the pandemic was a reminder of the historical atrocities inflicted by colonization and how they are healing from intergenerational historical trauma and will now have to heal from the outcomes of the pandemic.

You know, just maybe, maybe how are the communities dealing with their own historical trauma in know[ing] to heal from that? Because like I said, it’s kind of trickled down to our generation what happened back then. And so that’s what we’re dealing with and trying to heal from and then we’re going through this new thing of this pandemic, you know, not being able to do our ceremonies, not being able to connect with our own relatives.

One Indigenous recovery provider addressed the consequences of colonialism and the stigmatization of alcoholism among Indigenous Peoples. The provider described how they decolonize their work in recovery through mindful and intentional terminology, allowing clients to rewrite their narrative.

I love to let my clients identify their own process, and so because alcohol is extremely stigmatized in our community, like with… Native Americans in general, it’s super stigmatized…But in that way, I like to let my clients identify their process…I’ll ask them [to] define it, of course, with terminology and—because me, I identify as a person in recovery, because I consider myself an alcoholic still. I’ve been sober for six years…but if I went next door and drank one beer, I would drink 30 in a bottle. Like, I don’t think I would stop. And so for that reason, I’m still an alcoholic. I’m still recovering from alcohol. But for them, I let them say, what do you identify as? Because some don’t want to be called an alcoholic, they don’t want to be called the person in recovery, but you hear some pretty powerful responses. And some people would say, like one person said, the resiliency, they’re like, “I actually like to say I’m in resiliency”, you know, “OK, elaborate on that.” “Well, I’m in resiliency of something that’s destroyed my culture. I’m resilient. I’m in resilience. I’m defying alcohol.” And another person says, “I’m in victory. You know, I don’t like to say I’m in recovery. I’m in victory because I’m victorious over something every day that has destroyed my family and has killed family members. It still has affected my family”. All sorts of ways that people say they’re in a battle.

### 2.4. Theme 4: The Importance of Relationships

The fourth theme highlighted the importance of relationships in recovery. The centrality of interconnectivity and relationships is a shared foundational value among Indigenous communities, guided by the notion that our web of relations connects us with other people, all living beings, and the natural world. Based on the interviews with recovery providers and individuals in recovery, relationships were integral to their mental well-being. One participant stated,

I think we had to just go back to the family, they rely so heavily, heavily on their family structure. Almost across the board, they will go take care of the other family members who are sick, they’re very devoted and that’s just such a protective factor for them.

Due to the multigenerational nature of Indigenous families, providers acknowledged the strength of communal living and connectivity to family.

I think the positive thing is that they had a very large support system, whereas, like the majority of Americans felt very isolated and lost a lot during the pandemic.

One participant shared how their family’s teachings and traditional knowledge connected them with the universe through prayer, providing comfort and mental well-being.

My parents showed us pretty much we have our pollen hanging by the door and I try to walk in and do my prayer in the morning. That was really helpful and encouraging. You know, you’re going to be okay just to your prayers and let whatever come, comes. But you can do this… I am used to [doing] my morning prayers and then when the sun is setting, I do more offerings. So that was helpful in trying to reach out to the universe to keep me protected and my family… my siblings, my mom, my clients, you know, all those. I do offerings to kind of help me to have a mindset about. Being positive. And not live in fear so much.

## 3. Discussion

This qualitative study sought to understand the factors of resilience and mental well-being among members of the substance use recovery community, including providers and practitioners of Western-based addiction treatment services and Indigenous community members who themselves were in substance use recovery or engaged in addiction treatment during the COVID-19 pandemic. Our analysis identified four major themes, including (1) healthcare barriers, (2) culture in recovery, (3) the impact of colonization/historical trauma, and (4) the importance of relationships.

Participants recurrently spoke about the limited access to healthcare services. Indigenous communities are historically underserved concerning effective SUD treatment services and behavioral and mental health (Blume and Arthur 2021; Goodkind et al. 2010). The COVID-19 pandemic aggravated substance misuse and disrupted accessibility to SUD treatment services (Mellis et al. 2021), especially culturally responsive recovery services for Indigenous clients. Providers described the challenges of modifying their practices and transitioning to tele-behavioral health due to the COVID-19 pandemic. Frustration and feeling ill-equipped to support clients was a common complaint as recovery practitioners were overwhelmed by personal and professional demands (i.e., electronic communication with clients, death of their family members, death of family members of clients, death of clients, illness, and stress). Similarly, other studies have demonstrated how the COVID-19 pandemic impacted counseling services by hindering the mental, emotional, and physical capacity of mental health professionals themselves (Joshi and Sharma 2020).

Our findings also highlight the importance of culture in recovery. Despite the belief that culture can be used as a stand-alone modality for substance use treatment (Blume and Arthur 2021; Lavallee and Poole 2010; Gone and Trimble 2012; Gone 2013), our study demonstrated that the recovery providers viewed cultural practices as separate from the Western recovery treatments and counseling they provided. Examination of the results indicates several considerations for this outcome. Because most of our participant practitioners/providers identified as non-Indigenous, they may not have aligned with or fully understood the cultural values of their Indigenous clients. Additionally, all of our participant practitioners were likely trained in American/Western institutions that do not instruct on how to integrate culture into addiction and recovery treatment.

Though the recovery practitioners were familiar with Indigenous practices, and their interviews acknowledge that the community relies on ties to prayer and ceremony when traditional practices and teachings are referenced, it is not within the context of addiction treatment and counseling during the COVID-19 pandemic. Moreover, the cultural aspect of recovery may have been absent in treatment and counseling due to the state of emotional health and disruptive impacts on everyday life, impacting the recovery practitioners’ ability to include a cultural component in their recovery counseling/addiction treatment. While the inclusion of traditional practices and Indigenous culture in recovery treatment was not present, Indigenous clients emphasized the importance of cultural connectedness and their reliance on traditional activities to maintain health and wellness during the pandemic. This is a result that is supported in other Indigenous focused studies of SU/SUD treatment (Richer and Roddy 2023).

Participants also reported on the impact of historical trauma and colonization within recovery. Our findings are consistent with other research evidence of the adverse effects of historical trauma and colonization among Indigenous communities, contributing to significantly higher rates of SUDs (Skewes et al. 2024). The US government’s actions and policies aimed to eradicate Indigenous lifeways through cultural, spiritual, linguistic, and place/space displacement (Blume and Arthur 2021). The deliberate violence to solve the “Indian problem” led to trauma within Indigenous communities, which has been felt for generations (Brave Heart and DeBruyn 1998; Sotero 2006). With mottos of “kill the Indian, save the man” being initiated and objectives to assimilate Indigenous Peoples through Federal Indian Boarding schools, Indigenous children were stripped of their culture and identity (Brave Heart and DeBruyn 1998). This physical and cultural displacement has had a lasting impact on not only those who attended the boarding schools but also their parents, grandparents, and future children and grandchildren. While boarding schools are only one example of the callous attempts at Indigenous erasure, the consequences of colonialism persist intergenerationally, contributing to elevated substance use (Blume and Arthur 2021).

It is both ironic and intentional that the very policies that led to alcohol and substance abuse within Indigenous communities are the same policies that are now inhibiting substance use resources, education, and facilities that are necessary for recovery. Economic insecurity, low educational support and attainment, and infrastructure that fails to support the self-determination and sovereignty of Indigenous Nations have all stemmed from Federal policy and continue to contribute to historical trauma, substance use, and mistrust of the resources that are meant to treat them, including recovery treatments and counseling (Blume and Arthur 2021; Sotero 2006; Brave Heart and DeBruyn 1998).

Despite the challenges brought about by the COVID-19 pandemic, collective and historical trauma, and ongoing structural violence, participants found positive strategies to provide and continue down their path to recovery. For instance, participants engaged or maintained positive relationships to sustain their mental well-being and continue recovery efforts. Indigenous perspectives of community and relationships correlate with Western recovery services and treatments based on community, finding support from others in recovery, and sharing similar experiences (Best et al. 2025). Furthermore, while a gender-based or intersectional analysis was beyond the scope of this study, we acknowledge its importance. Prior research shows that gender, age, caregiving, and LGBTQ+ identities shape Indigenous recovery experiences and warrant further exploration (Gone and Trimble 2012; Rowan et al. 2014).

### Limitations and Strengths

There were multiple limitations of note. First, sample size was small and should be considered in the interpretation of the results. Second, though a purposive sampling method was used, participants were mainly comprised of recovery providers. Third, this study was conducted in one state in the Southwest US, restricting generalizability beyond this region. Fourth, while we used the collective term “recovery community” to honor the overlapping identities and shared lived experiences of our participants, we acknowledge providers that this decision limited our ability to disaggregate findings by subgroups such as recovery providers vs. individuals in recovery, Indigenous vs. non-Indigenous participants, or urban vs. rural-nation settings. This could have obscured meaningful differences in experience and perspective.

Multiple strengths offset these limitations. Namely, this tribal-university collaboration worked together with various stakeholders and members from each community to ensure that each stage of this study was community-informed and culturally congruent. Furthermore, the research team primarily consisted of Indigenous individuals, some of whom were from the participating Indigenous Nations. Our research team, comprised mostly of Indigenous researchers, helped build trust between the project team and the community and ensured that Indigenous perspectives were a part of the analysis and interpretation of the results.

## 4. Methods

### 4.1. Indigenous Guided Research

Our study employed an Indigenous lens through collaboration and consultation with relevant tribal research offices and community researchers. Out of respect for the requests of the participating communities, we do not disclose the specific communities or tribal research offices involved. Community researchers were Indigenous professionals living within or near the participating nations throughout the study. Tribal research offices refer to the different tribal IRB offices and/or research approval committees from each of the participating nations. The Indigenous community researchers, in collaboration with the Tribal research offices, played a critical role in every stage of the project, including study design, data collection, analysis, and the returning of findings and data back to each of the participating nations. We sought research approval from the appropriate tribal research entities for the three participating Indigenous nations. Our goal was to minimize the burden on our participants. We utilized talking circles for our group interviews to align with the cultural values of sharing and telling stories. For individual interviews, we were sensitive to ensuring our participants felt safe and had the opportunity to share what was important to them about their experience beyond our stated research script and questions. This article specfically focuses on findings from the individual interviews.

### 4.2. Relationships

Our research team was comprised of interdisciplinary researchers whose primary fields include Economics, Counseling Psychology, Applied Indigenous Studies, Public Health, and Indigenous Health. Each researcher brought their own Indigenous identities and knowledge to the project to elevate Indigenous voices and promote a narrative centered on local and cultural perspectives from the substance use recovery community. All authors identify as Indigenous Peoples and represent tribal identities with the Diné (Navajo) Nation, Ojibwe, Salish, and Choctaw.

### 4.3. Study Design and Participants

This study used qualitative analysis of transcribed narratives from interviews to understand the factors of resilience and mental well-being among members of the substance use recovery community, including providers and practitioners of Western-based addiction treatment services and Indigenous community members who themselves were in substance use recovery or engaged in addiction treatment during the COVID-19 pandemic. For further details about partnership development, study methods, recruitment, and research design, please refer to our protocol manuscript published elsewhere (Baldwin et al. 2023).

Participants were recruited from three participating Indigenous Nations in Arizona and an urban Indigenous-serving health organization. All participants were required to be 18 years and older and identify as Indigenous people in recovery or addiction treatment service providers of any identity but serving Indigenous people in recovery. We prioritized recruiting individuals who identified as American Indian/Native American/Indigenous and then requested any addiction treatment service providers to participate after a limited response to our outreach and engagement with AI recovery providers.

### 4.4. Data Collection

The research team adhered to COVID-19 safety guidelines from the Centers for Disease Control and Prevention (CDC) and conducted interviews virtually through Zoom or by telephone. Thus, we did not collect information on the participants’ physical location. Members of the recovery community research group (A.A.-J., C.K., J.R.) worked together to develop specific questions for the recovery participants to explore and understand the impacts of the COVID-19 pandemic on this population. In the individual interviews, we scheduled 90-min meetings but allowed participants to take the time they needed to share their stories. Interviews ranged from 90–180 min. In the individual sessions, we used a semi-structured interview guide, which included the following research questions: (1) Please describe to me how your work has changed because of the COVID-19 pandemic. (2) Please describe to me any special qualities or circumstances that have allowed Native Americans in recovery to adapt positively to the new circumstances presented by the presence of the virus. (3) Please describe to me any special qualities or circumstances that have affected Native Americans in recovery to negatively respond to the new circumstances presented by the presence of the virus. The semi-structured interview guide was used across all individual interviews to ensure consistency in the core research questions while allowing flexibility to adapt follow-up questions based on participants’ experiences and roles. This approach was intentional, as many participants held overlapping identities (e.g., both recovery providers and individuals in recovery), and the shared guide allowed us to capture a wide range of perspectives within the recovery community without reinforcing rigid distinctions. Further details on the study’s protocols can in the larger project team’s published manuscriipt (Baldwin et al. 2023).

### 4.5. Data Analysis

With participant permission, interviews were audio-recorded, transcribed verbatim, and uploaded into NVivo 12 (QRS International) for further analysis. A team of three Indigenous researchers analyzed the data, ensuring a thorough and collaborative approach to the coding process. The coding process was guided by the IDOH and Indigenous Nation Building frameworks, which shaped both the structure of our codebook and the interpretation of thematic content. In this study, resilience was not treated as a fixed trait but as a community-defined and culturally rooted process. As such, we operationalized resilience in our analysis through themes that reflected cultural connection, relational strength, and the ability to sustain recovery despite systemic barriers. For example, themes such as “Culture in Recovery” and “Importance of Relationships” were identified as core resilience pathways. These align with Indigenous models of wellness that recognize the role of teachings, spiritual practice, and kinship in promoting mental well-being.

Although our original interview guide did not explicitly use a strengths-based framework, participants frequently articulated protective factors through narratives of cultural survival, communal healing, and resistance to colonial impacts. These dimensions were captured in the coding and discussed as culturally embedded mechanisms of resilience. Guided by IDOH, which lays an Indigenous framework on the social determinants of health; and Indigenous Nation Building, a codebook was developed and used to conduct a thematic analysis to identify major themes (Baldwin et al. 2023). These frameworks were not used as broad concepts but were foundational to the study’s original design, developed in collaboration with the participating nations, and embedded throughout the research process. They directly informed the research questions, shaped our codebook, and guided the interpretation of themes. The diagram of the conceptual framework can be found in the previously published manuscript by Baldwin et al. (2023). The coding process involved an iterative approach, with researchers independently coding the transcripts, followed by team meetings to strategically refine and consolidate codes into themes. Furthermore, a team of Indigenous student workers, many from the participating Native Nations, assisted with quality checking the transcriptions prior to the thematic analysis.

## 5. Conclusions

Findings from our study capture a sliver of the challenges confronting Indigenous community members in recovery. Individuals in recovery, or providing recovery services, from each of the participating Indigenous Nations reported unique perspectives of their recovery practice during the COVID-19 pandemic. Given the impact of COVID-19, Indigenous client experiences of limited access to healthcare and recovery resources and the lack of culturally grounded treatment models are not recent disparities. AI/ANs are colonially disadvantaged. Many of the ongoing injustices and health inequities facing AI/AN communities are rooted in colonialism that systemically harms Indigenous Peoples. Our findings suggest the need for culturally grounded, community-driven recovery services that prioritize relationships and traditional practices. Policymakers and service providers should invest in Indigenous-led systems of care and support long-term strategies that address structural inequities rooted in colonization.

Despite these challenges, many participants shared how they or their clients leaned on positive relationships for support throughout the pandemic. Some also utilized cultural practices, though it was outside of recovery treatments and counseling. This demonstrated that regardless of the many challenges, including the shortcomings of the available recovery services, Indigenous communities, including the recovery community within, remained steadfast, identifying and using safe and positive strategies to maintain their health and mental well-being, and, in turn, resilience throughout the COVID-19 pandemic.

## Figures and Tables

**Table 1.** Participant Characteristics (*n* = 19).

Characteristics	Category	Number of Participants
Gender	Female	15
	Male	4
Ethnicity	Indigenous	15
	Non-Indigenous	4
Age Range	18–24	_-_
	25–34	1
	35–44	3
	45–54	9
	55–64	6
	65+	-

## Data Availability

The data sets generated and analyzed during this study are not publicly available due to confidentiality agreements with the respective Native Nations involved in the research. Data will be made available upon request, subject to approval from the respective nations. Please contact Dr. Julie A. Baldwin for further information.

## Abbreviations

AI/AN: American Indian/Alaskan Native
SU/SUD: Substance Use/Substance Use Disorder
IDOH: Indigenous Determinants of Health
